# Clinical characteristics, viral dynamics, and antibody response of monkeypox virus infections among men with and without HIV infection in Guangzhou, China

**DOI:** 10.3389/fcimb.2024.1412753

**Published:** 2024-06-24

**Authors:** Huiqin Yang, Xiaoqing Xie, Mou Zeng, Yinghui Cao, Qinghong Fan, Mengling Jiang, Chunliang Lei, Jian Wang, Feng Li, Xiaoping Tang, Haisheng Yu, Linghua Li

**Affiliations:** ^1^ Infectious Disease Center, Guangzhou Eighth People’s Hospital, Guangzhou Medical University, Guangzhou, China; ^2^ Institute of Infectious Diseases, Guangzhou Eighth People’s Hospital, Guangzhou Medical University, Guangzhou, China

**Keywords:** monkeypox virus, HIV, clinical specimens, viral load kinetics, men who had sex with men (MSM)

## Abstract

**Background:**

Monkeypox virus (MPXV) is spreading globally and nearly half of the infected people were human immunodeficiency virus (HIV) positive. Therefore, an in-depth understanding of the effects of HIV infection on the outcomes of MPXV infection is urgently needed. This study aimed to explore the clinical features, viral dynamics, and antibody response to MPXV infections in men who had sex with men (MSM) with and without HIV co-infection.

**Design or methods:**

MPXV-infected patients diagnosed by PCR were recruited in this study and were divided into MPXV and MPXV + HIV groups based on whether they were co-infected with HIV. Clinical data and samples were collected during of the hospital stay and follow up interviews. The symptoms and signs, laboratory examinations, viral shedding in various body fluids or swabs, antibody dynamics were tracked and compared between the two groups.

**Results:**

A total of 41 MPXV patients were recruited through June 2023 to September 2023 in Guangzhou. The MPXV group and MPXV + HIV group comprised 20 and 21 MSM, respectively. Patients in the two groups exhibited similar clinical characteristics except for pruritus and eschar, both were significantly fewer in MPXV + HIV group than in MPXV only group. Among the 355 clinical samples collected, MPXV DNA was detected in 100% of scabs, 97.4% of skin swabs, and 92.3% of exudate swabs from lesions, while the positive rate was 87.5% from oropharyngeal swabs, 59% from saliva, 51.3% from anal swabs, 50% from feces, 30.6% from urine samples, 37.5% of semen, and 28.2% from sera. Dynamics analysis revealed that viral DNA was undetectable in most patients 20 days after symptom onset. IgM and IgG antibodies to MPXV were detected in all patients with 3–5 days earlier in the MPXV group than in the MPXV + HIV group.

**Conclusion:**

This cohort analysis based on a large outbreak among MSM in Guangzhou indicated no obvious differences in clinical symptoms, viral DNA data, but antibody responses were 3–5 days later in mpox patients with HIV infection.

## 1 Introduction

The zoonotic monkeypox virus (MPXV) belongs to the genus *Orthopoxvirus* in the *Poxviridae* family (Lum et al., 2022). Genetically, MPXV can be divided into the West African (Clades II) and Central African (Clades I) clades. The latter clade has been associated with severe disease manifestations and increased infectiousness (Huang Y. et al., 2022). Interestingly, the monkeypox (MPOX) pathogen is characterized by limited human-to-human transmission. The cases reported outside Africa have been sparse and primarily linked to travel from regions endemic to MPOX (Tan and Gao, 2022). In May 2022, MPOX outbreaks surfaced in several non-endemic countries, predominantly in the Western countries. By May 2023, cases of this virus infections were recorded in China, especially Guangzhou, Beijing (Huang B. et al., 2022; Dou et al., 2023; Jiang et al., 2023; Yu et al., 2023). Notably, the MPXV were isolated from the 2022 outbreak belong to the B.1 lineage in China (Yu et al., 2023).

In the ongoing outbreak, approximately 95% of MPOX cases have been linked to sexual contact, and a recent study reported a staggering 98% transmission among men who have sex with men (MSM) (Thornhill et al., 2022). The MPXV transmitted via body fluids, mucous membranes, and compromised skin. Human-to-human transmission primarily occurs through close or indirect contact with virus-contaminated items (Zheng et al., 2023). The primary symptoms observed in MPXV infected individuals typically include fever, headache, lymphadenopathy, and a characteristic rash affecting the face and genitals (Palich et al., 2023; Suñer et al., 2023). Typically, MPOX is a self-limiting disease, presenting several symptoms and complications, such as secondary infections, proctitis, tonsillitis, bronchopneumonia, sepsis, encephalitis. Corneal infection can also happen, which could lead to vision loss (Stafford et al., 2023).

Almost half of patients were HIV positive in the ongoing worldwide MPOX epidemic (Thornhill et al., 2022; Palich et al., 2023). People living with human immunodeficiency virus (PLWH) with normal immune function displayed similar clinical presentations, complications and prognosis. A meta analysis indicated that PLWH had a higher occurrence of syphilis co-infection, skin rash, proctitis, cough and diarrhea. PLWH with a low CD4 count may face an elevated risk of severe outcomes due to MPXV infection, including a high prevalence of fulminant dermatological and systemic manifestations and death, especially with advanced or uncontrolled HIV infection (Curran et al., 2022; Shin et al., 2023). But the kinetic data of viral load on different clinical specimens and antibody response differences between PLWH and non-HIV MPXV patients were limited.

MPOX characteristics in China are distinctive. First, most cases include MSM due to community transmission (Dou et al., 2023; Zheng et al., 2023). Second, a significant proportion of cases come to attention during medical treatment, while only a small number are identified through the tracking and screening of close contacts (Liu et al., 2023). Third, most of the cases are characterized by clinical symptoms, including fever, herpes, and lymph node enlargement, with no severe cases or mortality. Lastly, many cases are identified through active consultation, whereas a small proportion is detected through close contact follow-up screening or active reporting and physical examination (CDC, 2023).

Although a self-limiting disease, MPOX continues to spread in specific populations. It has been transmitted continually since the concentrated outbreak in 2023; however, only a few cases have been reported. Global research endeavors are underway to enhance our understanding of the pathobiology and clinical characteristics of the disease. The investigative approach focuses on the unprecedented route of human-to-human transmission and the unique aspects of clinical presentation. Comprehensive data on viral shedding and antibody response dynamics will unravel the transmission and pathogenesis of MPOX and guide the development of diagnostic algorithms, surveillance strategies, and clinical management of this emerging global outbreak. In this study, we present the dynamics of viral release in various body fluids, the serum antibody profile post-infection, demographic and epidemiological characteristics, clinical manifestations, and health status of confirmed MPOX cases in Guangzhou, China.

## 2 Materials and methods

### 2.1 Study design and participants

This observational cohort study recruited all patients with MPXV infection confirmed by MPXV-specific polymerase chain reaction (PCR) and hospitalized at Guangzhou Eighth People’s Hospital of Guangzhou Medical University, from June 2, 2023 to September 1, 2023 (**Table 1**; **Supplementary Table 1**). The participants were divided into MPXV without HIV infection group (MPXV group) and MPXV with HIV infection group (MPXV + HIV group). Differences including clinical features, viral dynamics, and antibody response were analyzed between the two groups. Ethics approval for the laboratory tests related to this study was obtained from the Guangzhou Eighth People’s Hospital Ethics Committee (approval no. 202311248).

**Table 1:** Information of all patients with MPXV.

Characteristic	Overall(n=41)	MPXV (n=20)	MPXV+HIV(n=21)	*p value*
Gender (male,%)	41 (100)	20 (100)	21 (100)	1.00
Age(Median,IQR)	30 (26-34)	31.5 (28.8-37.5)	29.5 (25.8-33)	0.10
Smallpox vaccination (%)	0 (0)	0 (0)	0 (0)	–
Sexual contact (MSM,%)	41 (100)	20 (100)	21 (100)	1.00
CD4^+^T cell count (cells/μL)	804 (577-975)	804 (659-975)	825 (467-978)	0.52
CD8^+^T cell count (cells/μL)	1148 (818-1684)	968 (628-1220)	1385 (893-2263)	**0.04**
**Region of infection**				0.59
Chinese Mainland	34 (82.9)	17 (85)	17 (80.95)	
Hongkong	1 (2.4)	1 (5)	0 (0)	
Thailand	1 (2.4)	0 (0)	1 (4.76)	
NA	5 (12.2)	2 (10)	3 (14.29)	
Co-Infection				
HIV	21 (51.2)	0 (0)	21 (100)	–
Syphilis	18 (43.9)	5 (25)	13 (61.9)	**<0.001**
Genital herpes	3 (7.3)	2 (10)	0 (0)	–
Condyloma acuminatum	4 (9.8)	1 (5)	3 (14.29)	0.24
Others	5 (12.2)	2 (10)	3 (14.29)	0.56
Complication				
Non-complication	23 (56.1)	10 (50)	13 (61.9)	0.27
Proctitis	5 (12.2)	2 (10)	3 (14.29)	0.56
Edema penis	4 (9.8)	2 (10)	2 (9.52)	0.82
Skin soft tissue infection	2 (4.9)	0 (0)	2 (9.52)	0.15
Lymphadenitis	2 (4.9)	2 (10)	0 (0)	–
Pneumonia	1 (2.4)	0 (0)	2 (9.52)	0.15
Perianal abscess	5 (12.2)	0 (0)	5 (23.81)	**0.01**
Pharyngitis	1 (2.4)	1 (5)	0 (0)	–

Data are presented as case number (percentage %), median (P_25_-P_75_), or mean±SD.

P value was determined using the Chi-square test or Fisher’s exact test for categorical variables and the Mann-Whitney U test for continuous variables.

-, Statistics analysis is underpowered due to unsatisfied statistical criteria.

The bold indicates that the P value between the two groups is less than 0.05, and the difference is statistically significant.

### 2.2 Patient interviews and data collection

All participants agreed to interviews regarding their demographic, epidemiological, and clinical characteristics during hospital admission. Given that most patients presented to the hospital with rash symptoms following MPXV infection, the time point when the rash was identified was designated as day 0. Details of the number and location of MPOX lesions, systemic symptoms, and lymphadenopathies were recorded. From days 15–30 post-discharge, hospital doctors conducted telephone interviews with all of the participants to evaluate the clinical progression of symptoms and lesions. Some patients returned to the hospital for sample collection during follow-up.

### 2.3 Collection of clinical samples

Clinical samples were collected according to the “Guidelines for the Diagnosis and Treatment of MPOX (2022 edition)” (中国CDC, 2023). Trained medical staff collected samples from various sources, including skin lesions (vesicle fluid or dry scraping of scabs or scars), blood, and swabs of the oropharynx, rectum, and genitalia using standardized procedures, whereas the participants self-collected feces, urine, and semen samples following adequate training. The samples were preserved in a virus medium and transported to the laboratory within 2 h. The viral titer was estimated after heat-inactivation of the samples at 56°C for 30 min.

### 2.4 Quantitative PCR (qPCR) detection of MPXV

The MPXV PCR protocol involved the collection of clinical samples in virus collection tubes containing guanidine salt (CDRICH). Subsequently, 200 μL of samples were subjected to nucleic acid extraction using isolation kits (Daan Gene) on the Smart32 plus automatic nucleic acid extractor (Daan Gene). The extracted nucleic acid (5 μL) was then subjected to MPXV nucleic acid detection using fluorescent PCR (Daan Gene). The qPCR signal was detected on a Bio-Rad CFX 96-Deep Well equipment: initial denaturation at 95°C for 5 min, 45 cycles of denaturation at 95°C for 5 s and annealing/extension at 65°C for 35 s, and a final extension at 50°C for 2 min.

### 2.5 Detection of MPXV-specific antibodies

MPXV-specific antibodies were detected using ELISA. The plates were coated with 0.2 µg/mL A35 protein of MPXV (Sino Biological) in phosphate-buffered saline at 4°C for 16–18 h, washed with 0.1% PBST, and blocked with 0.5% bovine serum albumin at room temperature for 1 h. Subsequently, 100 μL of 10-fold serially-diluted and inactivated serum sample was added to the plates at 37°C for 30 min and incubated with 1:5,000 horseradish peroxidase-labeled goat anti-mouse IgG secondary antibody before 3,3′,5,5′-Tetramethylbenzidine detection. The absorbance measured at 450 nm on a Varioskan LUX plate reader (Thermo) was plotted against fold-dilution, and the AUC values were calculated to determine the intensity of each ELISA reaction.

### 2.6 Statistical analysis

Continuous variables were compared using the Mann–Whitney U test and are expressed as medians (interquartile range, IQR), whereas categorical variables were analyzed using the chi-square test or Fisher’s exact test and are represented as counts and percentages. *P* < 0.05 indicated a significant difference. All statistical analyses were performed using IBM SPSS version 25, and the results were represented graphically using GraphPad Prism 9.5.1 software.

## 3 Results

### 3.1 Clinical features of MPXV infection

The MPXV include 20 MSM and the MPXV + HIV groups include 21 MSM. All of the participants did not received smallpox vaccination. The median age of the cohort was 30 years old (IQR 26– 34). The CD4 count was 804 or 825 cells/mm^3^ in the MPXV or MPXV + HIV groups, respectively. Whereas the CD8 count was significantly lower in the MPXV group (Median: 968 cells/mm^3^) than in the MPXV + HIV group (Median:1,385 cells/mm^3^). Moreover, 5/20 (25%) infected individuals in MPXV group were syphilis-positive, while 13/21 (61.9%) were syphilis-positive in the MPXV+HIV group (**Table 1**; **Supplementary Table 1**).

**Figure 1:**
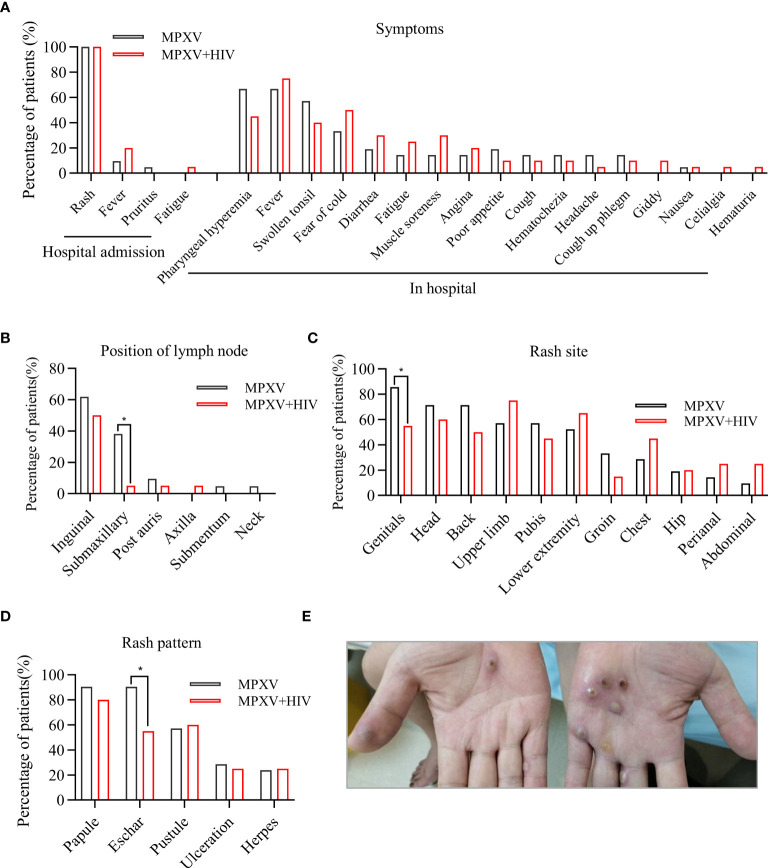
Clinical features of MPXV infection. **(A)** Clinical symptoms during hospital admission and hospital stay are compared between the MPXV and MPXV + HIV groups. **(B–D)** Analysis of rash symptoms and site in the MPXV (black) and the MPXV + HIV groups. **(E)** Image showing skin lesions with umbilicated papules on a patient’s hand with confirmed MPXV infection. * indicates that the P value between the two groups is less than 0.05, and the difference is statistically significant.

All patients exhibited skin lesions, with about 20% experiencing fever at the time of hospital admission. During hospitalization, patients showed symptoms of pharyngeal hyperemia, fever, and swollen tonsils without significant differences between groups (**Figure 1A**). Lymphadenopathy was observed in the inguinal region in all patients and in the submaxillary region in the MPXV group (**Figure 1B**). The patients displayed one or more lesion types with rash morphology including papules, eschar, and pustules (**Table 2**). 24/41 (58.5%) patients reported pruritus in their skin rashes, with 8 (38%) being HIV-positive individuals, significantly fewer than those without HIV (**Table 2**) HIV-positive patients exhibited less eschar patterns than the MPXV group (**Figures 1C–E**). Furthermore, 70%–85% of patients in the MPXV group presented rashes on the genitals, head, back, and pubis, and 70%–80% of the patients in the MPXV + HIV group had rashes on the upper limbs and lower extremities. Perianal rashes were observed in 5/21 (23.8%) MPXV + HIV patients, a higher prevalence than in the MPXV group (**Figure 1C**).

**Table 2:** Rash characteristics in all patients with MPXV.

Rash characteristics	Overall (n=41)	MPXV (n=20)	MPXV+HIV (n=21)	*p value*
Patients with rashes (n,%)	41 (100)	20 (100)	21 (100)	1.00
Rash number (Median,IQR)	8.5 (6-24.8)	9.5 (6-24.8)	7.5 (5.5-23.3)	0.98
Pruritus (n,%)	24 (58.5)	16 (80)	8 (38.1)	**<0.001**
Rash pain (n,%)	16 (39)	8 (40)	8 (38.1)	0.78

Data are presented as case number (percentage %), median (P_25_-P_75_), or mean±SD.

P values were determined using the Chi-square test or Fisher’s exact test for categorical variables and the Mann-Whitney U test for continuous variables.

-, Statistics analysis is underpowered due to unsatisfied statistical criteria.

The bold indicates that the P value between the two groups is less than 0.05, and the difference is statistically significant.

The counts and ratios of peripheral white blood cells, neutrophils, lymphocytes, monocytes, eosinophils, and basophils in both groups were within the normal range (**Table 3**). Also, no significant differences were observed in the coagulation indexes between the two groups, and the median values of PT, PTA, INR, TT, APTT, and Fibrin degradation products were within the normal levels (**Table 3**). However, the median values of D-dimer (1,180 µg/mL) in both groups exceeded the normal range. In addition, the immune-related serum amyloid A protein (SAA) (21.8 mg/L) level surpassed the normal range in both groups. The median values of C-reactive protein, interleukin (IL)-6, and IL-10 were within the normal range in the MPXV group but higher in the MPXV + HIV group (**Table 3**).

**Table 3:** Laboratory data from all patients with MPXV .

	Reference range	Overall (n=41)	MPXV (n=20)	MPXV+HIV (n=21)	*p value*
Blood routine examination					
WBC (10^9/L)	3.5-9.5	7.3 (5.9-8.9)	7.3 (5.8-8.5)	7.3 (6.4-8.9)	0.46
NEU (10^9/L)	1.8-6.3	3.8 (3.1-4.6)	3.7 (3.1-4.6)	3.8 (3.1-4.3)	0.81
NEU%	40.0-75.0	51.4 (44.7-62.8)	52.3 (47.1-62)	49.6 (43.8-62.8)	0.52
LYM (10^9/L)	1.1-3.2	2.7 (1.9-3.5)	2.6 (1.9-3)	2.9 (1.9-4.2)	0.40
LYM%	20.0-50.0	38.7 (27.3-45.8)	37.6 (27-41.3)	40.9 (27.9-48.1)	0.38
MONO (10^9/L)	0.1-0.6	0.5 (0.4-0.6)	0.5 (0.4-0.6)	0.6 (0.4-0.6)	0.72
MONO%	3,0-10.0	6.7 (5.6-7.9)	7.1 (5.8-7.9)	6.6 (5.3-7.8)	0.56
EOS (10^9/L)	0.02-0.53	0.2 (0.1-0.3)	0.2 (0.1-0.3)	0.2 (0.1-0.2)	0.25
EOS%	0.4-8.0	2.7 (1.6-3.9)	3.2 (2.1-4.2)	2.7 (1.4-3.1)	0.38
BASO (10^9/L)	0-0.06	0.02 (0.02-0.03)	0.02 (0.02-0.04)	0.02 (0.01-0.03)	0.21
BASO%	0-1.0	0.3 (0.2-0.5)	0.4 (0.2-0.5)	0.3 (0.2-0.4)	0.16
Blood coagulation test					
PT (seconds)	11.0-15.0	13.1 (12.9-13.5)	13.2 (12.9-13.7)	13.1 (12.9-13.4)	0.47
PTA (%)	70.0-120.0	100 (93-104)	99 (91.5-104)	100 (96-104)	0.54
INR	0.8-1.2	1 (1-1)	1 (1-1.1)	1 (1-1)	0.53
TT (seconds)	13.0-21.0	16.5 (16.2-17.1)	16.4 (16.1-16.9)	16.7 (16.2-17.1)	0.48
APTT (seconds)	28.0-43.0	39 (35.5-42.5)	40.8 (37-42.5)	38.5 (35.5-41.1)	0.40
Fib (g/L)	2.0-4.0	4.2 (3.5-4.5)	4.1 (3.4-4.7)	4.3 (3.9-4.5)	0.52
FDP (μg/mL)	<5.0	2.8 (2.2-3.5)	2.9 (2.3-3.3)	2.4 (2-3.7)	0.48
D-Dimer (μg/mL,DDU)	<1000	1180 (840-1545)	1180 (855-1527.5)	1135 (830-1545)	0.92
Immunity					
SAA (mg/L)	<10.0	21.8 (10.7-60)	20.3 (11.2-70.3)	21.8 (10.1-42.4)	0.61
CRP (mg/L)	<10.0	10.4 (9.9-34.2)	9.9 (9.9-33)	12.5 (9.9-34.2)	0.65
IL-2 (pg/mL)	0-5.71	0 (0-0.3)	0 (0-0.1)	0 (0-0.3)	0.45
IL-4 (pg/mL)	0-2.80	0.6 (0-1.1)	0.5 (0-1.1)	0.8 (0.3-1.4)	0.32
IL-6 (pg/mL)	0-5.30	4.4 (2.6-13)	3.6 (2.3-8.1)	7.9 (3.1-14.9)	0.11
IL-10 (pg/mL)	0-4.91	5.3 (4.2-8.4)	4.5 (3.8-7.3)	5.9 (4.8-9.9)	0.07
TNF-α (pg/mL)	0-4.60	1.2 (1-1.4)	1.3 (1.2-1.4)	1.2 (0.9-1.4)	0.18
IFN-r (pg/mL)	0-7.42	2.7 (1.3-6.1)	2.2 (1.3-5.7)	3.1 (1.6-6.2)	0.63

Data are presented as median (P_25_-P_75_).

P values were determined the Mann-Whitney U test for continuous variables.

### 3.2 Dynamics of MPXV DNA in each biological sampling site

**Figure 2:**
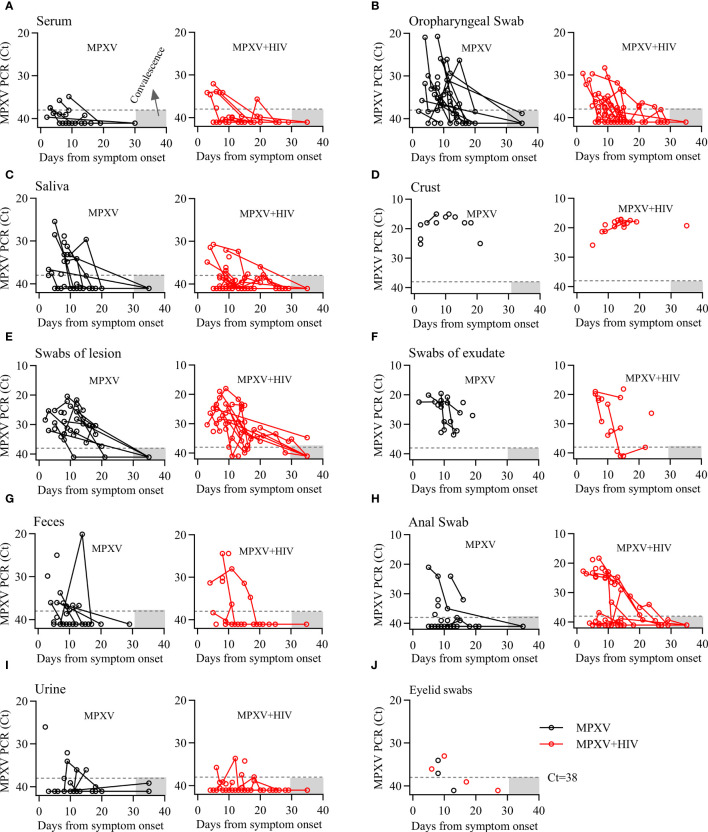
Dynamics of MPXV DNA shedding in different biological samples from the onset of the symptoms throughout infection. **(A–I)** Viral DNA levels in various longitudinal samples from patients with MPXV during infection. Viral DNA levels are presented as cycle threshold values (Ct). Sample collection days were adjusted for the onset of symptoms in each patient. Dashed lines indicate the limit of detection. Gray area represents the duration of follow-up.

The dynamics of MPXV DNA were investigated across 355 clinical samples collected from various body fluids or swabs post-hospital admission. The collection time point was dependent on clinical management. Various samples, including serum, saliva, crust, stool, urine, semen, and swabs of the oropharynx, skin lesions, exudate, stool, anus, and genitals, were collected at multiple time points from diagnosis throughout the disease and during follow-up. MPXV DNA was detected in several follow-up samples post-symptom onset: 11/39 (28.2%) serum, 35/40 (87.5%) oropharyngeal swabs (OPS), 23/39 (59%) saliva, 22/22 (100%) crust, 38/39 (97.4%) swabs of lesions, 24/26 (92.3%) swabs of exudate, 20/39 (51.3%) anal swabs, 17/34 (50%) stool, 11/36 (30.6%) urine, 3/8 (37.5%) semen, and 14/24 (9.5%) genital swabs. Notably, the positive rate and viral load of MPXV in anal swab were higher in HIV positive group than that in HIV negative group, whereas no significant differences were observed in other samples between the two groups (**Figures 2A–I**; **Table 4**).

**Table 4:** Summary of the MPXV DNA results in different samples collected from all patients with MPXV.

	Overall(n=41)	MPXV (n=20)	MPXV+HIV(n=21)	*p value*
Serum	11/39 (28.2)	6/20 (30)	5/19 (26.3)	0.80
Oropharyngeal Swab	35/40 (87.5)	19/20 (95)	16/20 (80)	0.34
Saliva	23/39 (59)	13/20 (65)	10/19 (52.6)	0.43
Crust	22/22 (100)	12/12 (100)	10/10 (100)	–
Swabs of lesion	38/39 (97.4)	19/19 (100)	19/20 (95)	1.00
Swabs of exudate	24/26 (92.3)	14/14 (100)	10/12 (83.3)	0.20
Anal swab	20/39 (51.3)	6/19 (31.6)	14/20 (70)	**0.02**
Feces	17/34 (50)	10/17 (58.8)	7/17 (41.2)	0.30
Urine	11/36 (30.6)	6/18 (33.3)	5/18 (27.8)	0.72
Semen	3/8 (37.5)	1/4 (25)	2/4 (50)	0.47
Genital swab	14/24 (58.3)	6/9 (66.7)	8/15 (53.3)	0.68

Data are presented as positive sample/collected sample (percentage %).

Ct value of less than 38 in nucleic acid testing is defined as positive.

P values were determined using the Chi-square test or Fisher’s exact test for categorical variables.

-, Statistics analysis is underpowered due to unsatisfied statistical criteria.

The bold indicates that the P value between the two groups is less than 0.05, and the difference is statistically significant.

The viral DNA level dynamics in various specimens from MPXV and MPXV + HIV-positive patients were shown in **Figure 2**. The MPXV DNA loads were <30 Cycle threshold (Ct30) in serum and urine, approximately Ct30 in OPS, saliva, and feces, and approximately Ct20 in crust samples. The swabs of the lesions and exudates exhibited Ct30–20 values. Serum was positive for MPXV within 10 days after symptom onset, whereas the other sample types exhibited a significantly decreased viral load in a time-dependent manner. Continues shedding of viral DNA was observed from most sampling sites, except for the crust, beyond 20 days from symptom onset. These findings expanded our understanding of MPXV shedding patterns in different body fluids, which could be helpful for assessing transmission risk and disease pathogenesis.

Some studies have shown that MPXV infection is associated with ocular manifestations (Cash-Goldwasser et al., 2022; Mazzotta et al., 2022). We also collected eyelid swabs from seven patients and found that four of them harboring low levels of viral DNA in early stages of infection but not during hospitalization (**Figure 2J**).

### 3.3 Antibody response during MPXV infection

**Figure 3:**
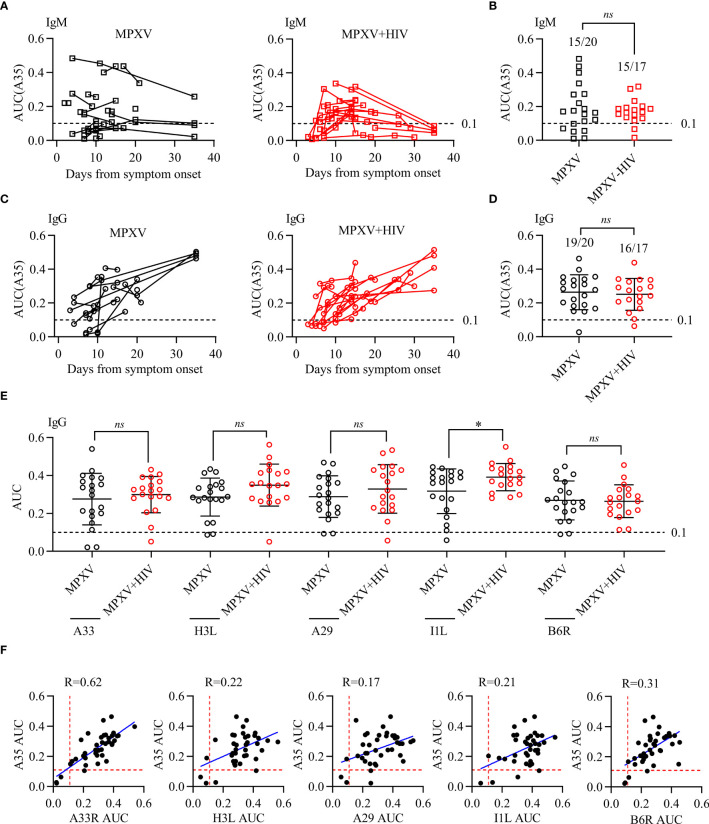
Antibody response to MPXV antigens during MPXV infection. Reactivity to MPXV antigens (A35, H3L, A29, I1L, and B6R) and vaccinia A33R was assessed using ELISA with 10-fold serial-diluted serum. ELISA OD450 data were plotted against fold-dilution to generate a reaction curve, and the AUC values for each ELISA curve were calculated to determine the reaction intensity. **(A, C)** IgM and IgG levels in response to A35 antigen detected in different longitudinal samples of patients during MPXV infection. **(B, D)** IgM and IgG levels against A35 antigen were tested at a selected point for each patient in different longitudinal samples of MPXV patients. **(E, F)** The correlation between A35 antibody levels and the other five antigens were analyzed to determine the specificity of these antigens. Dashed lines represent the detection limit of the AUC of ELISA, i.e., 0.1. * indicates that the P value between the two groups is less than 0.05, and the difference is statistically significant. ns means there is no significant difference between the two groups.

The present study measured the levels of anti-MPXV antibodies during infection in consecutive serum samples collected 2–40 days post-symptom onset (**Figure 3**). The serum levels of IgM and IgG to MPXV A35 antigen 37 patients were detected using ELISA. The area under the curve (AUC) for IgM was significantly higher in the early stages than at later time points, suggesting an early IgM response during MPXV infection (**Figure 3A**). In addition, 15/20 (75%) samples tested positive for anti-A35 IgM in the MPXV group, whereas 15/17 (88%) tested positive in the MPXV + HIV group (**Figure 3B**). Moreover, IgG levels increased gradually during the third week of symptom onset (**Figure 3C**). Throughout hospitalization, 95% of the samples were tested positive for antibodies against the A35 antigen (**Figure 3D**). Interestingly, the IgM and IgG responses appeared 3–5 days later in the MPXV + HIV group compared to the MPXV group. This delay could be attributed to compromised immune response due to HIV infection, although with combined anti-retroviral therapy.

Next, we examined four additional MPXV antigens (H3L, A29, I1L, and B6R) along with one vaccinia virus antigen (A33R) (**Figures 3E, F**). The positive rate of antibodies to these five antigens, determined by the cutoff value, was consistent with that of A35. In summary, these results indicated that MPXV infection triggers humoral immune response in all infected patients, with no significant differences between the MPXV only and MPXV + HIV groups.

In summary, antibody responses to MPXV infection were detected in all patients within one week of symptom onset. These antibodies were presented earlier in the MPXV group than in the MPXV + HIV group, highlighting the effect of HIV infection on antibodies response to MPXV.

## 4 Discussion

In the present study, we analyzed longitudinal data to characterize the clinical features and viral clearance time in outpatients with mild MPOX disease in a real-world. The results showed that most of the clinical characteristics differed insignificantly between the MPXV group and MPXV + HIV group, except for pruritus and the location of some skin lesions. Viral DNA remained detectable in swab samples for 10–20 days from the onset of symptoms. However, Viral DNA of most patients were no detectable after 30 days, except for the crust samples. Furthermore, the positive test for viral DNA is a limited indicator of infectiousness because PCR didn’t differentiate between viable and nonviable viruses. Based on our data, the viral DNA might take up to 30 days to reach undetectable levels in swabs and samples. Another major concern is whether MPXV could be transmitted through semen (Lapa et al., 2022; Reda et al., 2022). In this study, we detected MPXV DNA in semen samples from 3/8 (37.5%) patients and observed that positive PCR results may be insufficient to support the presence of viable viruses or infectivity. Additionally, our findings highlight that oropharyngeal and rectal samples exhibit a high positive rate of MPXV DNA and could be potential sources for MPXV detection.

MPOX disease might raise morbidity and cause prolonged illness in individuals with advanced HIV infection (CD4 < 350 cells/mm^3^) or with untreated HIV, resembling HIV-associated opportunistic infections (Zhou, 2023). However, in our cohort, four HIV-positive patients had CD4 counts <350 cells/mm^3^ but exhibited no characteristics of severe disease. None of the patients in our study had advanced acquired immunodeficiency syndrome, and they were relatively young, probably explaining the absence of severe disease. Thus, HIV-positive individuals should undergo comprehensive HIV testing and treatment to prevent MPXV infection or disease progression and reduce the risk of MPOX severity (Yinka-Ogunleye et al., 2023).

As the MPOX outbreak unfolded, initial findings suggested a widespread viral presence in both environmental and patient samples, creating an impression of abundance of the virus (Tarín-Vicente et al., 2022). However, recent studies have shown that some people contracted the disease from infected family members, even without sexual contact (similar infections were reported among close care workers in our hospital) (Salvato et al., 2022; Alarcón et al., 2023). This finding suggests that respiratory droplets and airborne particles may not be the primary transmission route (Peiró-Mestres et al., 2022; Thornhill et al., 2022). Also, the route of sexual transmission of the virus through blood, semen, or other bodily fluids during sexual activity remains uncertain. Conversely, another study highlighted that 95% of MPXV transmission occurs during sexual intercourse, involving 98% of the MSM community. Consequently, sexual activity among men has been identified as high-risk behavior (R0 > 1) (Cabanillas et al., 2023).

Previous findings and our data highlighted that patients with MPOX exhibit IgG and IgM responses as early as two days after the appearance of rash, and high levels of IgG antibodies can persist for more than three months (Karem et al., 2005). Consequently, IgG and IgM testing emerges as a valuable diagnostic tool, specifically in screening for asymptomatic transmission within the community or specific populations (Bhalla and Payam, 2023; Dai et al., 2023).

There are limitations about our study. First, the self-sampling strategy and minimizing loss to follow-up may have led to samples with lower accuracy than those collected by healthcare professionals. Second, because of the absence of a sensitive method for assessing MPXV viability in cell culture and the constraints of a biosafety level 3 laboratory, we could not determine whether samples with high viral DNA loads contained replication-competent viral particles. Third, the accuracy of the self-reported time points for the onset of skin lesion symptoms by patients upon admission may be compromised or biased. Lastly, since MPOX is a self-limiting disease primarily affecting young men, some patients were discharged without medical consent, leading to missing data and incomplete information.

## Data availability statement

The original contributions presented in the study are included in the article/**Supplementary Material**. Further inquiries can be directed to the corresponding authors.

## Ethics statement

The studies involving humans were approved by the ethical committee of Guangzhou Eighth People’s Hospital. The studies were conducted in accordance with the local legislation and institutional requirements. The participants provided their written informed consent to participate in this study.

## Author contributions

HYu: Conceptualization, Funding acquisition, Supervision, Writing – original draft, Writing – review & editing. HY: Investigation, Resources, Writing – original draft. XX: Data curation, Investigation, Methodology, Writing – original draft. MZ: Formal analysis, Investigation, Writing – original draft. YC: Data curation, Methodology, Writing – original draft. QF: Investigation, Resources, Writing – original draft. MJ: Investigation, Methodology, Writing – original draft. CL: Funding acquisition, Resources, Writing – original draft. JW: Resources, Writing – review & editing. FL: Funding acquisition, Resources, Writing – review & editing. XT: Funding acquisition, Resources, Writing – review & editing. LL: Resources, Validation, Writing – review & editing.
