# Supplementary material for: Clinical characteristics, viral dynamics, and antibody response of monkeypox virus infections among men with and without HIV infection in Guangzhou, China
Source: Front Cell Infect Microbiol. 2024 Jun 24;14:1412753. doi: 10.3389/fcimb.2024.1412753 (PMC11228139; doi:10.3389/fcimb.2024.1412753)
Supplement: Supplementary file 1 [file Presentation_1.pptx]

## Slide 1
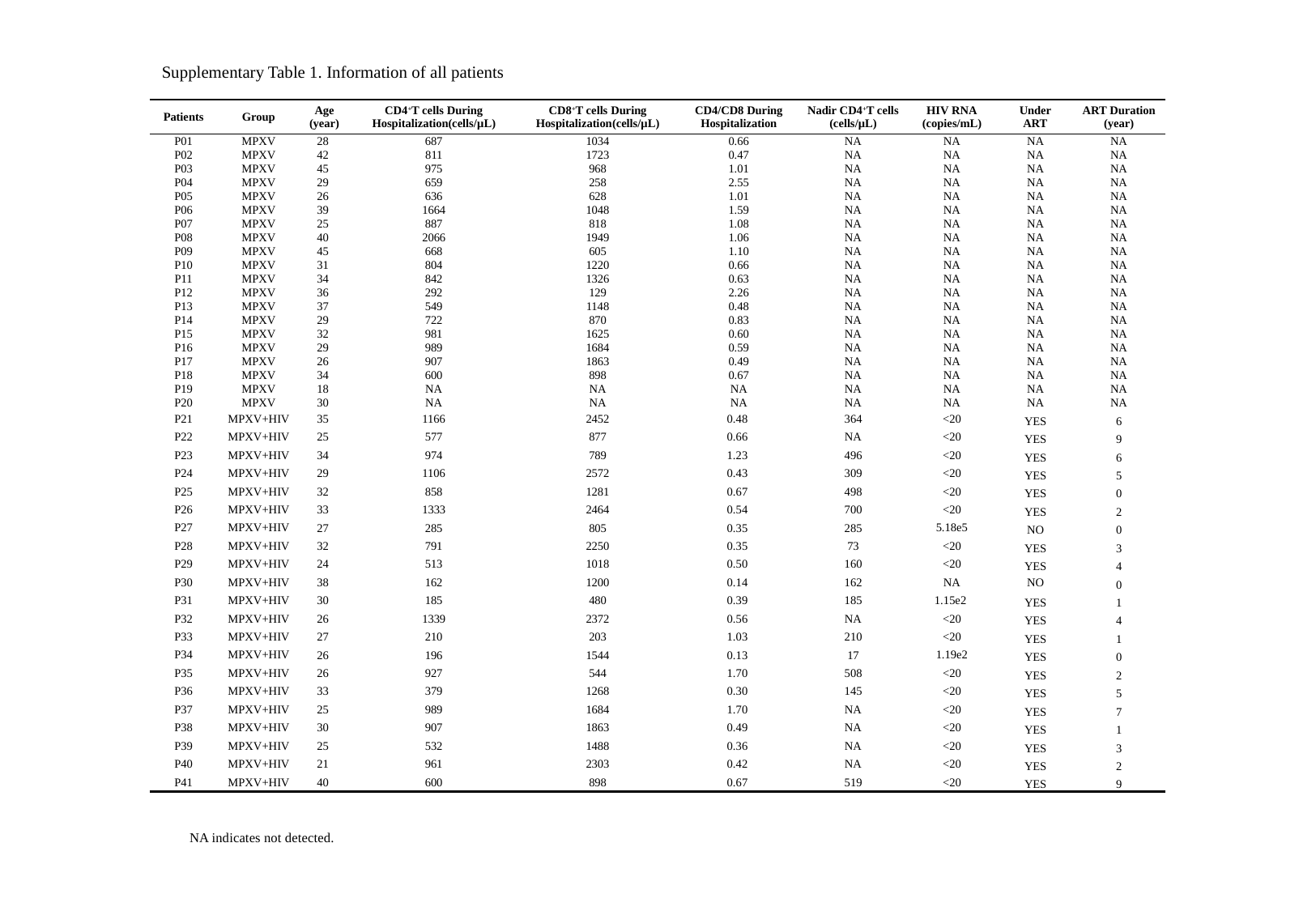

Supplementary Table 1. Information of all patients
| Patients | Group | Age (year) | CD4+T cells During Hospitalization(cells/μL) | CD8+T cells During Hospitalization(cells/μL) | CD4/CD8 During Hospitalization | Nadir CD4+T cells (cells/μL) | HIV RNA (copies/mL) | Under ART | ART Duration (year) |
| --- | --- | --- | --- | --- | --- | --- | --- | --- | --- |
| P01 | MPXV | 28 | 687 | 1034 | 0.66 | NA | NA | NA | NA |
| P02 | MPXV | 42 | 811 | 1723 | 0.47 | NA | NA | NA | NA |
| P03 | MPXV | 45 | 975 | 968 | 1.01 | NA | NA | NA | NA |
| P04 | MPXV | 29 | 659 | 258 | 2.55 | NA | NA | NA | NA |
| P05 | MPXV | 26 | 636 | 628 | 1.01 | NA | NA | NA | NA |
| P06 | MPXV | 39 | 1664 | 1048 | 1.59 | NA | NA | NA | NA |
| P07 | MPXV | 25 | 887 | 818 | 1.08 | NA | NA | NA | NA |
| P08 | MPXV | 40 | 2066 | 1949 | 1.06 | NA | NA | NA | NA |
| P09 | MPXV | 45 | 668 | 605 | 1.10 | NA | NA | NA | NA |
| P10 | MPXV | 31 | 804 | 1220 | 0.66 | NA | NA | NA | NA |
| P11 | MPXV | 34 | 842 | 1326 | 0.63 | NA | NA | NA | NA |
| P12 | MPXV | 36 | 292 | 129 | 2.26 | NA | NA | NA | NA |
| P13 | MPXV | 37 | 549 | 1148 | 0.48 | NA | NA | NA | NA |
| P14 | MPXV | 29 | 722 | 870 | 0.83 | NA | NA | NA | NA |
| P15 | MPXV | 32 | 981 | 1625 | 0.60 | NA | NA | NA | NA |
| P16 | MPXV | 29 | 989 | 1684 | 0.59 | NA | NA | NA | NA |
| P17 | MPXV | 26 | 907 | 1863 | 0.49 | NA | NA | NA | NA |
| P18 | MPXV | 34 | 600 | 898 | 0.67 | NA | NA | NA | NA |
| P19 | MPXV | 18 | NA | NA | NA | NA | NA | NA | NA |
| P20 | MPXV | 30 | NA | NA | NA | NA | NA | NA | NA |
| P21 | MPXV+HIV | 35 | 1166 | 2452 | 0.48 | 364 | <20 | YES | 6 |
| P22 | MPXV+HIV | 25 | 577 | 877 | 0.66 | NA | <20 | YES | 9 |
| P23 | MPXV+HIV | 34 | 974 | 789 | 1.23 | 496 | <20 | YES | 6 |
| P24 | MPXV+HIV | 29 | 1106 | 2572 | 0.43 | 309 | <20 | YES | 5 |
| P25 | MPXV+HIV | 32 | 858 | 1281 | 0.67 | 498 | <20 | YES | 0 |
| P26 | MPXV+HIV | 33 | 1333 | 2464 | 0.54 | 700 | <20 | YES | 2 |
| P27 | MPXV+HIV | 27 | 285 | 805 | 0.35 | 285 | 5.18e5 | NO | 0 |
| P28 | MPXV+HIV | 32 | 791 | 2250 | 0.35 | 73 | <20 | YES | 3 |
| P29 | MPXV+HIV | 24 | 513 | 1018 | 0.50 | 160 | <20 | YES | 4 |
| P30 | MPXV+HIV | 38 | 162 | 1200 | 0.14 | 162 | NA | NO | 0 |
| P31 | MPXV+HIV | 30 | 185 | 480 | 0.39 | 185 | 1.15e2 | YES | 1 |
| P32 | MPXV+HIV | 26 | 1339 | 2372 | 0.56 | NA | <20 | YES | 4 |
| P33 | MPXV+HIV | 27 | 210 | 203 | 1.03 | 210 | <20 | YES | 1 |
| P34 | MPXV+HIV | 26 | 196 | 1544 | 0.13 | 17 | 1.19e2 | YES | 0 |
| P35 | MPXV+HIV | 26 | 927 | 544 | 1.70 | 508 | <20 | YES | 2 |
| P36 | MPXV+HIV | 33 | 379 | 1268 | 0.30 | 145 | <20 | YES | 5 |
| P37 | MPXV+HIV | 25 | 989 | 1684 | 1.70 | NA | <20 | YES | 7 |
| P38 | MPXV+HIV | 30 | 907 | 1863 | 0.49 | NA | <20 | YES | 1 |
| P39 | MPXV+HIV | 25 | 532 | 1488 | 0.36 | NA | <20 | YES | 3 |
| P40 | MPXV+HIV | 21 | 961 | 2303 | 0.42 | NA | <20 | YES | 2 |
| P41 | MPXV+HIV | 40 | 600 | 898 | 0.67 | 519 | <20 | YES | 9 |
NA indicates not detected.
